# Long-Term Visual Outcomes for a Treat-and-Extend Antivascular Endothelial Growth Factor Regimen in Eyes with Neovascular Age-Related Macular Degeneration: Up to Seven-Year Follow-Up

**DOI:** 10.1155/2020/3207614

**Published:** 2020-07-31

**Authors:** Simon Javidi, Ali Dirani, Fares Antaki, Marc Saab, Sofiane Rahali, Ghassan Cordahi

**Affiliations:** ^1^Centre Universitaire d'Ophtalmologie, Hôpital Maisonneuve-Rosemont, Université de Montréal, Montréal, QC, Canada; ^2^Centre Universitaire d'Ophtalmologie, Hôpital du Saint-Sacrement, CHU de Québec, Université Laval, Québec, QC, Canada; ^3^Département d'ophtalmologie, Hôpital Charles-Le Moyne, Université de Sherbrooke, Greenfield Park, QC, Canada

## Abstract

**Purpose:**

To report long-term visual and anatomical outcomes in eyes with neovascular age-related macular degeneration (nAMD) treated with a treat-and-extend regimen (TER) of intravitreal antivascular endothelial growth factor (anti-VEGF) injections in real-world settings.

**Methods:**

Retrospective cohort study of consecutive patients with nAMD treated with a TER of anti-VEGF intravitreal injections by a single retina specialist (GC). Patients with nAMD who had at least one year of follow-up were identified using an electronic database. Best-corrected visual acuity (BCVA), comprehensive ophthalmologic examination, and macular OCT were performed at each visit. Patients received a loading dose of three monthly intravitreal injections and then were treated according to a TER of bevacizumab, ranibizumab, and/or aflibercept. The number of injections, BCVA, and central retinal thickness (CRT) were evaluated during the follow-up period.

**Results:**

180 eyes from 180 patients were included in the study. Mean age was 75 ± 9 (range: 51–96). Mean BCVA was 0.77 ± 0.64 LogMAR at baseline, 0.69 ± 0.58 LogMAR (*p* = 0.0057) after loading phase, 0.64 ± 0.55 LogMAR (*p* = 0.0001) after 6 months of TER, and 0.76 ± 0.71 LogMAR after 6 years of treatment (*n* = 32 at year 6). CRT decreased significantly after the loading phase (*p* = 0.0002). The mean number of intravitreal injections per year was 7.6 during the first three years of treatment and then decreased to 5.9 during year 4 to 7.

**Conclusions:**

This retrospective study of 180 nAMD patients treated with a TER of intravitreal anti-VEGF demonstrates an initial improvement of BCVA after loading phase, followed by long-term visual stabilization for at least six years. These results were obtained with a high number of injections, averaging close to six injections per year during long-term follow-up. In light of the natural evolution of nAMD, these data support the long-term efficacy of this treatment under real-world conditions of heterogeneity of patients and type of anti-VEGF used.

## 1. Introduction

Age-related macular degeneration (AMD) is a major cause of visual impairment and blindness in the elderly population [1]. It is responsible for 46% of cases of severe visual loss in patients over the age of forty [2]. Neovascular AMD (nAMD) occurs in only 10% of patients with AMD but is responsible for most cases of blindness [3]. The management of nAMD has seen a tremendous breakthrough with the introduction of intravitreal (IVT) anti-VEGF injections: pegaptanib sodium in 2004, off-label bevacizumab in 2005, ranibizumab in 2006, and aflibercept in 2011 [4].

In 2006, the ANCHOR and MARINA trials demonstrated the safety and efficacy of ranibizumab in nAMD compared to sham and verteporfin photodynamic therapy [5, 6]. Subsequently, bevacizumab was shown to be noninferior to ranibizumab in terms of efficacy in the IVAN and CATT trials [7, 8]. Aflibercept injected monthly or every two months, after a loading dose of 3 monthly injections, was also shown to be noninferior to the monthly regimen of ranibizumab in the VIEW 1 and 2 trials [9, 10]. Anti-VEGFs have since become the first-line of treatment in most cases of nAMD.

However, these pivotal trials were based on monthly injections, which in real-life long-term settings are of significant burden for patients, caregivers, healthcare practitioners, and healthcare systems [11]. Alternative treatment regimens therefore emerged. The PrONTO prospective study in 2009 introduced the Pro-Re-Nata (PRN) regimen consisting of a loading dose of 3 consecutive monthly injections, followed by monthly visits with OCT-guided retreatment based on disease activity. After 2 years of PRN regimen, they achieved similar visual outcomes in comparison to monthly injections, but with fewer intravitreal injections [12]. However, subsequent studies observed that the PRN regimen may not offer the same results demonstrated in the PrONTO study [13, 14].

Real-life data issued from the landmark trials for nAMD treatment was examined in the SEVEN-UP study [15]. Long-term outcomes from the ANCHOR and MARINA trials could not be extrapolated from the 2-year results and frequent injection was found to be needed in order to preserve visual acuity in the long run [15]. The need for an alternative treatment regimen offering adequate outcomes while requiring less frequent visits was evident; the idea of an individualized strategy known as “treat-and-extend regimen (TER)” was introduced in 2007 by Richard Spaide [16]. TER consists of a loading phase of 3 monthly injections, followed by a maintenance phase where patients are given an injection at each visit and treatment interval is gradually extended or shortened, based on the absence or presence of disease activity [17]. Several trials have since demonstrated the efficacy of TER, but visual acuity outcomes remained inferior to the data obtained in the original landmark randomized clinical trials [18–20].

With the intent of promoting data-driven practices, we conducted this study to assess the real-world long-term outcomes of intravitreal anti-VEGF treatment based on the TER in patients with nAMD.

## 2. Methods

This study was designed as a retrospective cohort study of consecutive patients followed and treated for nAMD by a single retina specialist (GC) at a single private retina practice in Montreal, QC, Canada, between 2009 and 2017. Patients included in the study were those with a diagnosis of nAMD who were receiving anti-VEGF injections on the basis of the TER and who had at least 12 months of follow-up after their first injection at our clinic; we excluded patients that did not have a proper loading phase (defined as less than 3 monthly injections within a timeframe of up to 18 weeks) or that were not compliant to TER during the first year of follow-up and treatment (noncompliance was defined as a missed visit, with a delay superior to one month in the subsequent visit after the missed visit). Patients were not excluded on the basis of noncompliance after the first year of follow-up and treatment. All patients were older than 50 years with either a newly diagnosed treatment-naïve nAMD or a previously treated nAMD. Patients with visual acuity worse than 20/320 (Snellen) were also included in the study, in contrast to the MARINA trial [6]. In patients with bilateral disease at first visit, we randomly selected which eye to include in the study. Exclusion criteria included choroidal neovascularization (CNV) secondary to other maculopathies, diabetic retinopathy, vein occlusions, and inflammatory maculopathies. Patients previously treated elsewhere using photodynamic therapy, intravitreal steroids, or thermal laser were not excluded. Patients with other common ocular comorbidities such as cataract or glaucoma were also not excluded. Data was sampled at baseline visit, month 3 (after loading phase), month 6, year 1, and then every “6 months” after that. Of note, some patients had few visits per year and therefore would have a visit not exactly at midyear or beginning of a year; in those cases, we attributed that data to the closest date it would correspond to, that is, either midyear or beginning of a year. In a minority of cases, patients had so few visits that data sampling at a specific timepoint did not occur although the patient's data was still sampled at the precedent and following timepoint (but with no visit/data in between). Also, in order to be eligible for the number of injections per year and anti-VEGF agent used per year analysis, only complete years of follow-up were considered; in situations where patients had a follow-up of, for instance, six years and a few months, only the complete six years were presented in those analysis, and the remaining months of the incomplete year seven were discarded (no extrapolations were made).

We identified 186 patients eligible for participation in the study. Six patients were excluded because of incomplete charts due to concomitant follow-up elsewhere. Ultimately, 180 eyes from 180 patients were included in the study. Data extracted from the charts included baseline characteristics such as demographics (age and sex) as well as past ocular history (lens status, history of glaucoma or pars plana vitrectomy). Information from the initial ophthalmic visit and subsequent follow-ups was also recorded, including involved eye, BCVA (Snellen), the intravitreal anti-VEGF agent injected (bevacizumab, ranibizumab, or aflibercept), central retinal thickness (CRT) as seen on OCT, and ophthalmic adverse events. The local research department confirmed that no ethical approval was required given the retrospective nature of this study, as there was no deviation from the usual standard of care. This study was conducted in concordance with the World Medical Association Declaration of Helsinki.

In order to better study the obtained data, we divided the cohort in two subgroups according to the treatment status at initial presentation: the “treatment-naïve” subgroup consisted of eyes with no previous nAMD treatment prior to first injection at our clinic, and the “previously treated” subgroup contained eyes that had received previous nAMD treatment (including prior anti-VEGF injections) prior to first injection at our clinic.

### 2.1. Treatment Regimen

Patients underwent an initial loading phase of 3 consecutive monthly injections. Subsequently, injections were given on a monthly basis until disease stability. The treatment intervals were then extended by 2 weeks per interval, up to a maximum of 12 weeks. If there were signs of recurrent disease at a given follow-up, (1) if the dosing interval was 6–8 weeks, the interval was decreased by 2 weeks and (2) if the dosing interval was 10–12 weeks, the interval was decreased by 4 weeks. This algorithm would be followed until resolution of recurrent disease. On the second attempt at extending, if disease instability occurred at the same interval as the previous recurrence, no further attempt was made to extend, and the last stable interval was maintained assuming disease stability. Disease instability was defined as new or persistent haemorrhage, intra- or subretinal fluid on OCT or leakage on fluorescein angiography (FA); FA was performed when available and not on a routine basis. Our definition of disease stability is absence of disease instability. In cases of severe recurrences, particularly if associated with new haemorrhages, the treatment interval would immediately be reduced back to monthly injections.

### 2.2. Choice of Anti-VEGF Agent and Injection Technique

Patients were treated with 0.5 mL IVT injections of either bevacizumab 1.25 mg (Avastin®), ranibizumab 0.5 mg (Lucentis®), or aflibercept 2.0 mg (Eylea®). These drugs were obtained commercially. Careful aseptic technique was used to fill the syringes directly from the vial. Topical anesthesia with proparacaine hydrochloride (0.5%) and asepsis with 5% povidone-iodine solution were applied prior to injections. Injections were performed 3.5 to 4.0 mm posterior to the limbus in the inferotemporal quadrant. The choice of anti-VEGF agent was guided by Dr. GC, based on his discretion and on the provincial funding for anti-VEGF agents. The decision to switch from one anti-VEGF agent to another was mostly based on the persistence of CNV activity (intra- or subretinal fluid) despite six consecutive monthly injections.

### 2.3. Optical Coherence Tomography (OCT)

OCT imaging was performed on all patients. Between 2009 and 2017, the OCT machines used were the CIRRUS 5000 machine (Carl Zeiss Meditec, Jena, Germany) and the Nidek RS-3000 (Nidek, Gamagori, Aichi, Japan). Eyes with available central retinal thickness (CRT) (measured using the map provided with the OCT software) were included in the analysis of CRT through the follow-up period. Adjustments were made to correct for different types of machines by converting CRT from Nidek RS-3000 (RS) to Cirrus (CR) equivalent using the following formula: CR = 8.00 + 1.01 × RS.[21]. In 2016, a data loss occurred in the Nidek machine leading to significant loss of CRT data from 2009 to 2016. Patients with no baseline CRT were therefore excluded from the CRT analysis since their baseline OCTs were not available.

### 2.4. Statistical Analysis

**Figure 1:**
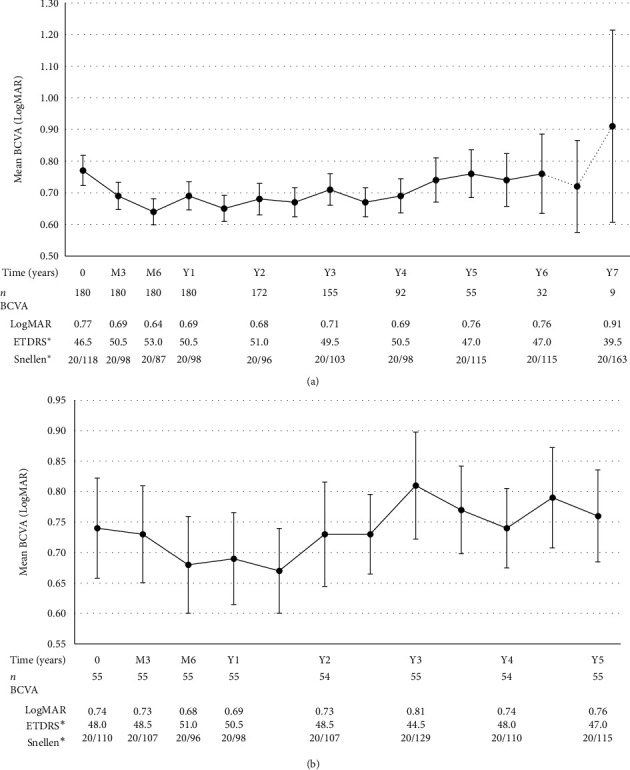
(a) Long-term mean BCVA (all patients). (b) Mean BCVA in subgroup of patients with at least 5 years of follow-up. Note: variance is expressed in form of standard error of the mean; data depicted as dotted lines must be interpreted with caution as it represents a sample size of *n* < 30. ^∗^ETDRS and Snellen equivalents were calculated from LogMAR values.

**Figure 2:**
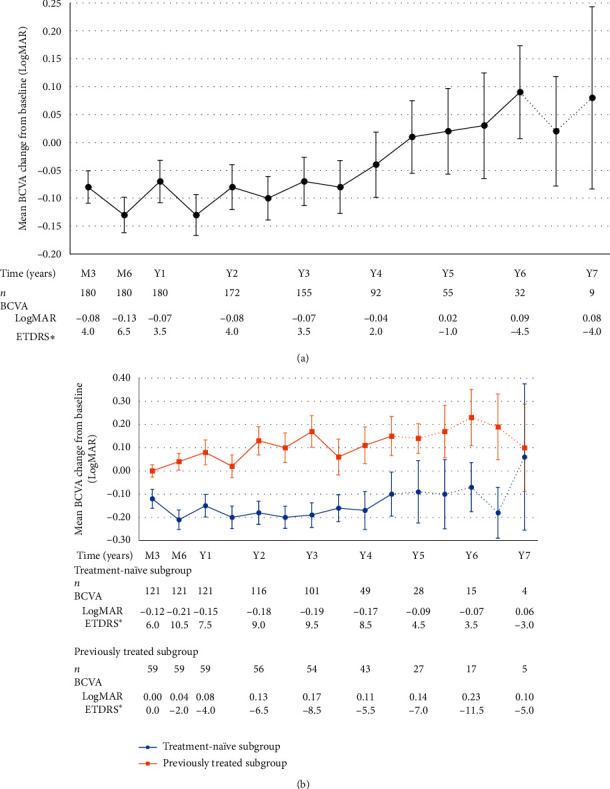
(a) Long-term mean BCVA change from baseline. (b) Mean BCVA change from baseline according to previous treatment status. Note: variance is expressed in form of standard error of the mean; data depicted as dotted lines must be interpreted with caution as it represents a sample size of *n* < 30. ^*∗*^ETDRS and Snellen equivalents were calculated from LogMAR values.

The primary outcome measures were BCVA over time and the number of injections per year, following the first injection, from years one to seven. Secondary outcomes included the anti-VEGF agent used and CRT. BCVA was measured on an imperial scale (Snellen) and converted into LogMAR for statistical analysis [22]. LogMAR visual acuities were also converted to equivalent ETDRS letter scores to illustrate the distribution of change in BCVA from baseline [23]. Of note, in Figures 1 and 2, we also presented BCVA data in ETDRS equivalent ± Snellen equivalent to ease interpretation for readers [24]. Baseline demographics were summarized by presenting the number and percentage for categorical variables and the average ± standard deviation (SD) for continuous variables. The association between variables was tested using unpaired and paired *t*-test for continuous variables with a parametric distribution. For continuous variables with a nonparametric distribution, the Mann–Whitney *U* and Wilcoxon signed-rank tests were used. Because the duration of follow-up was heterogeneous within the cohort due to different inclusion timepoints for each patient, the analysis was performed at regular intervals: 3 months, 6 months, 1 year, and every six months after. Differences with a *p* value less than 0.05 were considered statistically significant. *p* values were not adjusted for multiple comparisons. Statistical analysis was conducted using Microsoft Excel (Microsoft Corp., Redmond, WA).

## 3. Results

### 3.1. Baseline Demographic Characteristics

One hundred eighty participants (180 eyes) were included in this study. The mean follow-up per patient was 4.0 ± 1.5 years (range: 1 to 7 years) during the study period.  Table 1 illustrates baseline characteristics of patients prior to first injection at the clinic.

There were 105/180 (58.3%) female patients. The mean age was 75 ± 9 with ages ranging from 51 to 96. Regarding past ocular history, 92/180 eyes (51.1%) were pseudophakic, 15/180 (8.3%) had glaucoma and 1/180 (0.6%) had previous pars plana vitrectomy surgery.

Although 121/180 (67.2%) eyes were treatment-naïve at baseline, 59/180 (32.8%) had a history of past nAMD treatment(s): 58/180 (32.2%) IVT anti-VEGF injection(s), 1/180 (0.6%) IVT corticosteroid injection(s), and 3/180 (1.7%) argon laser for extrafoveal CNV. None had previous photodynamic therapy.

Eye involvement at baseline was as follows: 146/180 (81.1%) patients had nAMD in a single primary eye at initial presentation and 34/180 (18.9%) patients had bilateral disease at initial presentation.

### 3.2. Baseline Ophthalmological Parameters

Mean BCVA and mean CRT at baseline were compared between two subgroups based on treatment status. In treatment-naïve eyes, the mean BCVA was 0.83 ± 0.64 LogMAR compared to 0.64 ± 0.60 LogMAR in the previously treated eyes: there was no statistically significant difference in BCVA at baseline (*p* = 0.0689). Similarly, no statistically significant difference was found between those subgroups in terms of CRT (*p* = 0.7141), as demonstrated in Table 2.

### 3.3. Number of Injections per Year

Table 3 illustrates the number of injections per year in the total cohort and compares the previously treated and treatment-naïve subgroups. For the total cohort, the mean number of injections per year was 9.1 ± 2.2 for the first year, 7.1 ± 2.4 for the second year, 6.7 ± 2.9 for the third year, and an average of 5.9 from years 4 to 7.

We compared the treatment-naïve and the previously treated subgroups. Both subgroups required a high number of injections during their first year of treatment and there was no statistically significant difference in the number of injections between the subgroups at that timepoint (*p*=0.5055). During the two subsequent years, a statistically significant difference in the number of injections was noted: on average, the previously treated subgroup required 0.9 (at year 2) and 1.2 (at year 3) more injections than the treatment-naïve subgroup (*p*=0.0372 and *p*=0.0116, respectively). There were no statistically significant differences between these subgroups from years 4 to 7.

### 3.4. Visual Outcomes

Table 4 demonstrates the evolution of BCVA across selected timepoints. Mean BCVA improved significantly during the first year of treatment. Mean BCVA at month 3, month 6, and year 1 was compared to baseline BCVA. BCVA improved from 0.77 ± 0.64 LogMAR to 0.69 ± 0.58 LogMAR after the loading phase at month 3 (*p* = 0.0057), 0.64 ± 0.55 LogMAR at month 6 (*p* = 0.0001), and 0.69 ± 0.60 LogMAR at year 1 (*p* = 0.0585). Mean BCVA improvement from baseline was significant during year 2 (*p* < 0.05) and thereafter regressed close to baseline values from years 3 to 6 (there was no statistically significant difference between the mean BCVA at each timepoint compared to baseline from years 3 to 6). This is illustrated further in Figure 1(a). We conducted a subanalysis of a subgroup of patients that had at least 5 years of follow-up (*n* = 55), in order to present their BCVA evolution over 5 years which is illustrated in Table 5 and Figure 1(b): this subgroup of patients had an initial improvement of BCVA within the first year of treatment (although not statistically significant), and overall, baseline BCVA was maintained over the course of 5 years.

Mean BCVA change from baseline was −0.08 ± 0.39 LogMAR at month 3, −0.13 ± 0.43 LogMAR at month 6, and −0.07 ± 0.51 LogMAR at year 1. Mean BCVA change from baseline went from −0.04 ± 0.56 LogMAR at year 4 (*n* = 92) to 0.02 ± 0.57 LogMAR at year 5 (*n* = 55). Although this change was not statistically significant, it demarcated the point in our observational study where mean BCVA change from baseline shifted from an improvement to a deterioration. At year 6, mean BCVA change from baseline was 0.09 ± 0.47 LogMAR (*n* = 32), which is not statistically significant (*p* = 0.3081). Beyond month 78, sample size decreases from 26 to 9 patients, which hinders further analysis. This is also illustrated in Figure 2(a). We conducted a subanalysis comparing BCVA change from baseline of treatment-naïve and previously treated subgroups. This subanalysis demonstrated that mean BCVA change from baseline was overall significantly better in the treatment-naïve subgroup in comparison to the previously treated subgroup, results of which are illustrated in Table 6 and Figure 2(b).

**Figure 3:**
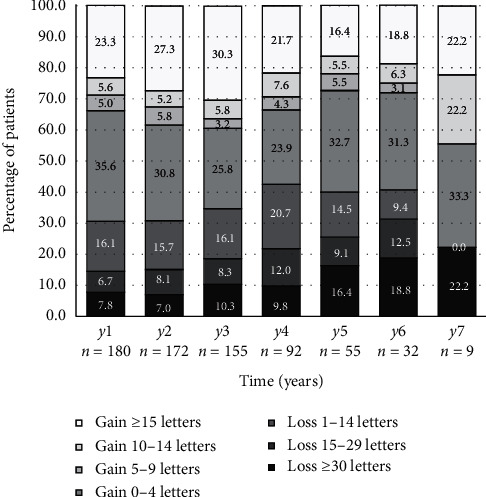
Distribution of changes in BCVA from baseline (equivalent ETDRS letter score). Note: ETDRS equivalent was calculated from LogMAR values.

Figure 3 illustrates the distribution of change in equivalent ETDRS score from baseline across selected timepoints. At year 1 (*n* = 180), 42/180 patients (23.3%) gained ≥15 ETDRS letters, 125/180 (69.4%) gained or maintained vision (≥0 ETDRS letters), and 154/180 (85.6%) were considered to have stabilized disease (less than 15 ETDRS letter loss), and only 26/180 (14.4%) lost ≥15 ETDRS letters. At year 6 (*n* = 32), 6/32 (18.8%) gained ≥15 ETDRS letters, 19/32 (59.4%) gained or maintained vision, 22/32 (68.8%) were considered to have stabilized disease, and 10/32 (31.3%) lost ≥15 ETDRS letters. The results from the remaining years are summarized in Figure 3.

### 3.5. Anatomical Outcomes

Table 4 also illustrates the evolution of mean CRT across selected timepoints. Mean CRT improved significantly in the first year of treatment. Mean CRT at month 3, month 6, and year 1 was compared to baseline CRT. Mean CRT improved from 402 ± 194* μ*m to 313 ± 140* μ*m after the loading phase at month 3 (*p* = 0.0002), 286 ± 102* μ*m at month 6 (*p* = 0.0001), and 313 ± 143* μ*m at year 1 (*p* = 0.0007). Mean CRT change from baseline was −86 ± 152* μ*m at month 3, −116 ± 164* μ*m at month 6, and −91 ± 175* μ*m at year 1. Mean CRT improvement from baseline was considered statistically significant until year 2. This improvement did not maintain statistical significance at year 3.

### 3.6. Type of Anti-VEGF Used

**Figure 4:**
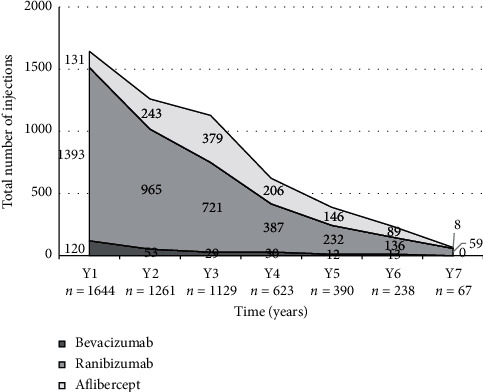
Type of anti-VEGF agent used per year.

Figure 4 highlights the types of anti-VEGF drugs used in the study. A total of 5352 IVT injections were given throughout the course of the study: 3893/5352 (72.7%) ranibizumab, 1202/5352 (22.5%) aflibercept, and 257/5352 (4.8%) bevacizumab. Most eyes received more than one type of anti-VEGF agent throughout the study period; only 47/180 (26.1%) of eyes received strictly one type of anti-VEGF.

We conducted a subanalysis of eyes that were switched from an anti-VEGF agent to another due to the persistence of CNV activity (intra- or subretinal fluid) despite six consecutive monthly injections. There was a total of 55 switches, 48/55 (87.3%) of which mostly occurred in the first three years of treatment. Most of the switches (46/55 (83.6%)) consisted of a transition from ranibizumab to aflibercept.

### 3.7. Loss to Follow-Up and Adverse and Surgical Events

Eighteen patients (10%) were lost to follow-up during the seven years of follow-up, distributed evenly throughout the seven years mostly secondary to very poor visual prognosis, relocation, death, or unknown reasons. Of note, other than these eighteen patients, the decline in sample size over the course of the study is due to the fact that total follow-up per patient was not even, as it ranged from 1 year to 7 years of follow-up. Adverse and surgical events that could have impacted BCVA are recorded in Table 7. During the course of the study, three cases of endophthalmitis occurred out of a total of 5352 injections (0.056%). 41/180 (22.8%) patients underwent cataract surgery and 12/180 (6.7%) had YAG-laser capsulotomy. Seven patients required pars plana vitrectomy (PPV) for the following reasons: 3/180 (1.7%) for injection related endophthalmitis, 2/180 (1.1%) for injection related retinal detachment, and 2/180 (1.1%) for vitreous haemorrhage. Of note, one patient underwent a second PPV for silicone oil removal following initial PPV for RD; these two surgeries took place within the same BCVA/CRT sampling interval. We conducted a subanalysis comparing BCVA prior to versus after cataract surgery: mean BCVA change after cataract surgery was −0.15 ± 0.29 LogMAR (*p* = 0.0005); 19/41 (46.3%) patients demonstrated an improvement of BCVA after cataract surgery.

## 4. Discussion

The advent of anti-VEGF revolutionized the treatment of nAMD. Despite ongoing efforts to find an ideal treatment regimen that balances visual outcomes and patient burden, the gold standard has not yet been established. Several studies like the TREX-AMD (2017), TREND (2018), and CANTREAT (2019) trials demonstrated noninferiority of ranibizumab with TER versus monthly regimen in treatment-naïve eyes, in patients treated up to 1 year [25–27]. Similarly, systematic reviews by Gemenetzi and Patel, Rufai et al. and Okada et al. suggested that TER is superior to PRN and comparable to monthly injections in the short term and highlighted the need for more real-word long-term data [20, 28, 29]. Of note, although we present statistically significant change in BCVA as *p* value <0.05 (cf. Table 4), we personally prefer looking at data from Figure 3 and conclude that patients either had disease stabilization or not: based on Figure 3, patients who had less than 15 ETDRS letters (3 lines) loss in comparison to baseline were essentially considered to have “disease stabilization,” and those that did not were considered to be progressing [15, 30].

Our study was a retrospective cohort study presenting real-world longitudinal data on patients with nAMD treated with anti-VEGF injections based on the TER. We included 180 eyes from 180 patients and reported visual acuity and anatomic outcomes as well as data regarding the number of injections per year. Our population was well-balanced at baseline and baseline BCVA and CRT was comparable among treatment-naïve and previously treated patients.

In our study, the mean number of injections per year for the whole cohort was higher in the first year of treatment (9.1 ± 2.2). The mean number of injections steadily declined over the next years and on average, the patients received close to six injections per year between years 4 and 7. We compared treatment-naïve and previously treated eyes and demonstrated that although a high number of injections is required in both subgroups in the first year of treatment, treatment-naïve eyes required on average less injections long-term. The difference was statistically significant at years 2 and 3. The most commonly used agent in our study was ranibizumab (72.7%), consistent with current trends on first-line choice of drug in Canada, as demonstrated by the CAN-PAT survey [31].

In 2017, Berg et al. published long-term follow-up data on TER for nAMD. Their patients required an average of 6.4 injections per year over the course of 7 years for treatment-naïve eyes [32]. Their result is consistent with our findings for treatment-naïve eyes as our subanalysis demonstrates an average of 6.5 injections per year in the same time frame. Mekjavic et al. and Khanani et al. also reported similar results over the course of 5 years, with an average of 6.1 and 6.3 injections per year, respectively, in their treatment-naïve eyes [33, 34]. Interestingly, in their cohort of 210 eyes, Mrejen et al. reported a higher number of injections (mean of 8.3 injections per year over the course of 6 years) in eyes with similar baseline characteristics. A possible reason why Mrejen et al. reported a higher number of injections is the inclusion of bilateral eyes at baseline where the worse eye dictates the visit interval for the other eye that could require less visits; they report a 13.5% rate of bilateral disease at the study inclusion timepoint [35].

In regard to visual acuity outcomes, data from BCVA change from baseline demonstrated a mean improvement of −0.08 LogMAR following the loading phase at month 3 and −0.13 LogMAR at month 6, results that were statistically significant. The statistically significant improvement from baseline BCVA was mostly maintained until the end of year 2 (except at timepoint year 1: *p* = 0.0585). From years 3 to 4, mean BCVA change from baseline demonstrated a sustained mean improvement (<0.00 LogMAR), although not considered statistically significant. From years 5 to 7, mean BCVA change from baseline demonstrated a slight worsening (>0.00 LogMAR) in comparison to baseline values, which was not considered statistically significant. In our subanalysis comparing treatment-naïve eyes versus previously treated eyes, we demonstrated that treatment-naïve eyes had significantly better BCVA outcomes.

Other studies have demonstrated an overall similar trend in visual acuity outcomes regarding mean BCVA change from baseline. Berg et al. reported an improvement of −0.11 LogMAR reaching −0.17 LogMAR at year 2. Despite a decrease in the amplitude of improvement following year 2, the statistically significant improvement was maintained until year 4. Subsequently, between years 6 and 7, visual acuity started deteriorating below baseline, although not considered statistically significant. They hypothesized that this long-term decline in vision could be explained by macular atrophy [32]. In Mrejen et al.'s cohort study, the visual acuity changes followed a similar tendency. There was an improvement in year 1 (−0.09 LogMAR) and year 2 (−0.11 LogMAR) followed by BCVA stabilization in the subsequent years. Similar to our results, Mekjavic and Zaletel Benda reported a BCVA improvement that peaked at year 1 followed by stabilization until year 5. Like us, they noted that their mean BCVA change from baseline shifted above 0.00 LogMAR between year 4 and year 5, although not statistically significant [33].

Khanani et al. included 93 eyes with good baseline BCVA (20/20–20/60) and showed that 65/93 (69.9%) of eyes had a BCVA equal or better than baseline at year 1, and 15/26 (57.7%) at year 5 [30]. We obtained very similar results in our study: 125/180 (69.4%) of eyes had a BCVA equal or better than baseline at year 1 and 33/55 (60.0%) at year 5. This is further illustrated in Figure 3. In addition, Figure 3 highlights the clinical significance of our results beyond statistical significance: the majority of our patients were considered to have sustained or improved BCVA at all timepoints from year one to seven, and in the long run, only less than a third of patients did not have “disease stabilization” (less than 15 ETDRS (3 lines) loss in comparison to baseline), which supports the long-term efficacy of anti-VEGFs under TER in real-world settings.

In regard to anatomical outcomes, we demonstrated a reduction in CRT. There was a statistically significant decrease of −91* μ*m at year 1. The improvement was maintained during the following year. This seems in line with the findings obtained by Berg et al. [32].

Additionally, 41/180 (22.8%) of patients in our study underwent cataract surgery (Table 7), which might impact BCVA outcomes, but we didn't exclude these patients since cataract progression and surgery is part of real-world conditions in the nAMD patient population. In our subanalysis comparing BCVA prior to versus after cataract surgery, only 19/41 (46.3%) patients demonstrated an improvement of BCVA after cataract surgery, but mean BCVA change after cataract surgery was −0.15 ± 0.29 LogMAR (*p*=0.0005), which corresponds to a mean improvement of 7.5 equivalent ETDRS letters after cataract surgery. Of note, Mrejen et al. found no statistically significant association between BCVA and cataract surgery in nAMD patients under long-term real-world conditions; 30/210 (14.3%) of eyes had cataract surgery over the course of their 6 year study [35]. Nonetheless, data is ambiguous on this topic, and a more recent study from Kessel et al. focused on this subject specifically and concluded that cataract surgery improved BCVA by an average of 7.1 ETDRS letters 6 months after surgery in nAMD patients with a mean BCVA of 52 ETDRS letters prior to surgery; their data reflects our findings [36].

Furthermore, 7/180 (3.9%) eyes underwent pars plana vitrectomy for complications related to nAMD or its treatment, but these patients were not excluded from our study, as we strived to represent real-world outcomes of this disease. Despite these limitations, our study provides robust statistically and clinically significant conclusions that add to the body of knowledge on real-world data for TER in the treatment of nAMD. We report 3/5352 (0.056%) cases of endophthalmitis, which is within norms, but slightly higher than the latest literature on this subject, as the pooled endophthalmitis rate from 20 large retrospective studies on IVT anti-VEGF injections was reported to be 144/510,396 (0.028%) [37].

Our study has some limitations intrinsic to its retrospective nature and real-world setting. There is lack of information on baseline data on nAMD duration in previously treated eyes and potential demographic confounders (e.g., education status, socio-economic status, marital status, access to relatives/help for visits, etc.). Also, data sampling not occurring exactly at the defined timepoints is a limitation. Excluding patients that were noncompliant to TER in the first year of follow-up and treatment must be taken into account as it has an impact on external validity. Conversion of BCVA from Snellen to LogMAR and ETDRS is another limitation. The choice of the initial drug was not independent of baseline characteristics and may have been a confounder for the number of injections and visual outcomes; the choice of the initial drug was at the discretion of the retina specialist and depended on the provincial funding for anti-VEGF agents. Mixing different anti-VEGF agents could also be a confounder, but it corresponds to the real-life practice of many clinicians. Progression of cataract and cataract surgery is also a confounder to be taken into account in such studies. In cases where patients had bilateral disease, the eye requiring a shorter interval of injection according to TER dictated the interval for the other eye that could have possibly required less frequent visits for injections under TER, which nonetheless is inherent to real-world settings. Intrinsic to the retrospective nature of the study, the number of patients decreased significantly after year 6 and data beyond that timepoint must be interpreted with caution. Of note, all of our patients have free coverage for anti-VEGFs in Canada; insurance coverage is therefore not a limitation in this study.

To conclude, this retrospective study of 180 eyes with nAMD treated with intravitreal anti-VEGF injections using the TER demonstrates an initial improvement of visual outcomes during the first few years of treatment, followed by visual stabilization for up to 7 years for the majority of our patients. This study also highlights the need for a high number of visits/injections per year (roughly 6 injections per year) throughout long-term follow-ups under the real-life conditions of the TER in nAMD. Overall, we demonstrated long-term efficacy of this treatment in real-world conditions: heterogeneity of patients, occasional struggles with visit compliance, various types of anti-VEGFs used, and so on. Our results are comparable to similar long-term real-world studies on the TER in nAMD.

## Figures and Tables

**Table 1:** Baseline demographics of patients and eyes.

Total number of patients/eyes	180
*Age*	
Mean (years) ± SD	75 ± 9
Range (years)	51–96
*Sex, n (%)*	
Male	75 (41.7%)
Female	105 (58.3%)
*Laterality of disease at first visit, n (%)*	
Unilateral	146 (81.1%)
Bilateral	34 (18.9%)
*Past ocular history, n (%)*	
Pseudophakic	92 (51.1%)
Glaucoma	15 (8.3%)
Pars plana vitrectomy	1 (0.6%)
*Past nAMD treatment, n (%)* ^*∗*^	
None	121 (67.2%)
Anti-VEGF	58 (32.2%)
Intravitreal corticosteroid	1 (0.6%)
Argon laser for extrafoveal CNV	3 (1.7%)
Photodynamic therapy	0 (0.0%)

^*∗*^Categories not mutually exclusive, except for “Past nAMD treatment: None”.

**Table 2:** Baseline ophthalmological characteristics.

	Total number of patients (BCVA: *n* = 180, CRT: *n* = 56)	Treatment status^*∗*^		
		Treatment-naïve subgroup (BCVA: *n* = 121, CRT: *n* = 48)	Previously treated subgroup (BCVA: *n* = 59, CRT *n* = 8)	*p* value
Mean BCVA (LogMAR)	0.77 ± 0.64	0.83 ± 0.64	0.64 ± 0.60	0.0689
Mean CRT (*μ*m)	402 ± 194	406 ± 188	378 ± 241	0.7141

^*∗*^Definitions: treatment-naïve eyes = no previous nAMD treatment prior to first injection at our clinic; previously treated eyes = previous nAMD treatment prior to first injection at our clinic.

**Table 3:** Number of injections per year in the total cohort and among the treatment-naïve and previously treated subgroups.

Timepoint	All eyes		Treatment-naïve subgroup		Previously treated subgroup		*p* value
	*n*	Number of injections, mean ± SD	*n*	Number of injections, mean ± SD	*n*	Number of injections, mean ± SD	
Year 1	180	9.1 ± 2.2	121	9.2 ± 2.2	59	9.0 ± 2.3	0.5055
Year 2	178	7.1 ± 2.4	120	6.8 ± 2.5	58	7.7 ± 2.2	0.0372
Year 3	172	6.7 ± 2.9	116	6.3 ± 2.9	56	7.5 ± 2.7	0.0116
Year 4	102	6.2 ± 2.8	56	6.3 ± 2.7	46	6.2 ± 3.1	0.8632
Year 5	72	5.6 ± 3.1	38	5.6 ± 3.3	34	5.1 ± 3.0	0.9684
Year 6	42	5.7 ± 2.8	24	5.3 ± 3.0	18	6.2 ± 2.5	0.2736
Year 7	11	6.1 ± 4.2	6	5.7 ± 4.5	5	6.6 ± 4.3	0.7344

**Table 4:** Evolution of BCVA and CRT across selected timepoints.

Timepoint		Visual acuity					Anatomical outcomes			
Year	Month	*n*	BCVA, mean (LogMAR) ± SD	BCVA change from baseline, mean (LogMAR) ± SD	*p* value^*∗*^	*n*		CRT, mean (*μ*m) ± SD	CRT change from baseline, mean (*μ*m) ± SD	*p* value^*∗*^
	0	180	0.77 ± 0.64	—	—	56		402 ± 194	—	—
	3	180	0.69 ± 0.58	−0.08 ± 0.39	0.0057	49		313 ± 140	−86 ± 152	0.0002
	6	180	0.64 ± 0.55	−0.13 ± 0.43	0.0001	45		286 ± 102	−116 ± 164	0.0001
1	12	180	0.69 ± 0.60	−0.07 ± 0.51	0.0585	48		313 ± 143	−91 ± 175	0.0007
	18	173	0.65 ± 0.54	−0.13 ± 0.48	0.0007	49		302 ± 136	−89 ± 168	0.0005
2	24	172	0.68 ± 0.65	−0.08 ± 0.53	0.0485	51		306 ± 155	−107 ± 210	0.0006
	30	171	0.67 ± 0.60	−0.10 ± 0.51	0.0105	42		304 ± 175	−105 ± 204	0.0018
3	36	155	0.71 ± 0.62	−0.07 ± 0.54	0.1360	35		354 ± 237	−52 ± 203	0.1345
	42	135	0.67 ± 0.53	−0.08 ± 0.55	0.0831	33		324 ± 182	−64 ± 198	0.0743
4	48	92	0.69 ± 0.52	−0.04 ± 0.56	0.4534	4		283 ± 85	−106 ± 255	—^*∗∗*^
	54	82	0.74 ± 0.63	0.01 ± 0.59	0.8189	—		—	—	—
5	60	55	0.76 ± 0.56	0.02 ± 0.57	0.7782	—		—	—	—
	66	50	0.74 ± 0.59	0.03 ± 0.67	0.7383	—		—	—	—
6	72	32	0.76 ± 0.71	0.09 ± 0.47	0.3081	—		—	—	—
	78	26	0.72 ± 0.74	0.02 ± 0.50	0.7795	—		—	—	—
7	84	9	0.91 ± 0.91	0.08 ± 0.49	—^*∗∗*^	—		—	—	—

^*∗*^ *p* value for BCVA/CRT change at different timepoints in comparison to baseline BCVA/CRT. ^*∗∗*^Sample size too small to conduct statistical analysis.

**Table 5:** Evolution of BCVA in subgroup of patients that completed at least 5 years of follow-up.

Timepoint		Visual acuity			
Year	Month	*n*	BCVA, mean (LogMAR) ± SD	BCVA change from baseline, mean (LogMAR) ± SD	*p* value^*∗*^
	0	55	0.74 ± 0.61	—	—
	3	55	0.73 ± 0.59	−0.01 ± 0.41	0.9228
	6	55	0.68 ± 0.59	−0.06 ± 0.46	0.3548
1	12	55	0.69 ± 0.56	−0.05 ± 0.52	0.5114
	18	54	0.67 ± 0.51	−0.07 ± 0.49	0.3014
2	24	54	0.73 ± 0.63	−0.01 ± 0.48	0.8651
	30	54	0.73 ± 0.48	−0.02 ± 0.46	0.7739
3	36	55	0.81 ± 0.65	0.08 ± 0.49	0.2489
	42	54	0.77 ± 0.53	0.03 ± 0.53	0.6794
4	48	54	0.74 ± 0.48	0.00 ± 0.50	0.9708
	54	53	0.79 ± 0.60	0.06 ± 0.57	0.4329
5	60	55	0.76 ± 0.56	0.02 ± 0.57	0.7782

*Note. n* = 55 patients completed at least 5 years of follow-up; 28/55 (50.9%) treatment-naïve, 27/55 (49.1%) previously treated. ^*∗*^*p* value for BCVA change at different timepoints in comparison to baseline BCVA.

**Table 6:** Comparison of BCVA change from baseline between the treatment-naïve subgroup and previously treated subgroup.

Timepoint		Treatment-naïve subgroup		Previously treated subgroup		*p* value
Year	Month	*n*	BCVA change from baseline, mean (LogMAR) ± SD	*n*	BCVA change from baseline, mean (LogMAR) ± SD	
	3	121	−0.12 ± 0.45	59	0.00 ± 0.20	0.0466
	6	121	−0.21 ± 0.46	59	0.04 ± 0.28	0.0001
1	12	121	−0.15 ± 0.53	59	0.08 ± 0.41	0.0041
	18	116	−0.20 ± 0.52	57	0.02 ± 0.37	0.0037
2	24	116	−0.18 ± 0.54	56	0.13 ± 0.46	0.0002
	30	116	−0.20 ± 0.51	55	0.10 ± 0.47	0.0002
3	36	101	−0.19 ± 0.53	54	0.17 ± 0.50	0.0001
	42	87	−0.16 ± 0.54	48	0.06 ± 0.53	0.0217
4	48	49	−0.17 ± 0.57	43	0.11 ± 0.52	0.0155
	54	44	−0.10 ± 0.63	38	0.15 ± 0.52	0.0595
5	60	28	−0.09 ± 0.71	27	0.14 ± 0.33	0.0444
	66	26	−0.10 ± 0.76	24	0.17 ± 0.55	0.1556
6	72	15	−0.07 ± 0.41	17	0.23 ± 0.50	0.0414
	78	12	−0.18 ± 0.38	14	0.19 ± 0.53	0.0512
7	84	4	0.06 ± 0.63	5	0.10 ± 0.42	—^*∗*^

^*∗*^Sample size too small to conduct statistical analysis.

**Table 7:** Noteworthy events and possible confounders throughout study duration.

Timepoint	*n*	Cataract surgery	YAG-laser capsulotomy	Pars plana vitrectomy	Endophthalmitis	Retinal detachment
Year 1	180	6	4	1	1	0
Year 2	178	12	4	2	0	2
Year 3	172	9	1	2	1	0
Year 4	102	8	2	2	1	0
Year 5	72	6	0	0	0	0
Year 6	42	0	1	0	0	0
Year 7	11	0	0	0	0	0
Total	180	41	12	7	3	2

## Data Availability

The database used to support the findings of this study may be provided upon request, by contacting the corresponding author.
